# Coincident Pellucid Marginal Degeneration and Fuchsiridocyclitis: a case report

**DOI:** 10.1016/j.ijscr.2025.112029

**Published:** 2025-10-07

**Authors:** Ali Hendi Alghamdi

**Affiliations:** a Unit of Ophthalmology, Department of Surgery, Faculty of Medicine, Al-Baha University, Al-Baha, 65431, Saudi Arabia

**Keywords:** Pellucid Marginal Degeneration, Fuchs' heterochromic iridocyclitis, Corneal ectasia, Uveitis, Cataract surgery, Glaucoma

## Abstract

**Introduction & importance:**

We report a rare coexistence of Pellucid Marginal Degeneration (PMD) and Fuchs' heterochromic iridocyclitis (FHI) in a 40-year-old female. The overlap of corneal ectasia and chronic uveitis created significant diagnostic and surgical challenges, highlighting the importance of individualized planning in complex anterior segment diseases.

**Case presentation:**

The patient presented with painless, progressive visual decline in the right eye. Clinical examination revealed inferior corneal thinning, mature cataract, pigmented keratic precipitates, and peripheral anterior synechiae (PAS). Corneal tomography confirmed PMD with a characteristic “crab-claw” pattern. The patient underwent phacoemulsification with posterior chamber intraocular lens implantation. Postoperatively, visual acuity improved, although optic disc cupping and persistent PAS underscored the need for long-term glaucoma surveillance.

**Clinical discussion:**

The coexistence of PMD and FHI is exceptionally rare. PMD complicates surgical planning by affecting keratometric reliability and corneal biomechanics, while FHI contributes to cataract formation, intraocular inflammation, and risk of secondary glaucoma. Multimodal imaging, tailored surgical strategy, and vigilant postoperative follow-up are key to optimizing outcomes.

**Conclusion:**

Simultaneous PMD and FHI represent a unique clinical entity requiring comprehensive assessment and individualized management. Tailored surgical planning and vigilant surveillance are essential to preserve vision and mitigate long-term risks.

## Highlights

• Coexistence of pellucid marginal degeneration (PMD) and Fuchs' uveitis presenting for cataract surgery
• Pre-operative tomography and angle assessment guided intraocular lens (IOL) strategy in an ectatic cornea.
• Intra-operative observation of the Amsler sign with implications for glaucoma surveillance
• Post-operative visual and refractive outcomes presented with time-stamped follow-up

## 1 Introduction

Pellucid Marginal Degeneration (PMD) is a rare, bilateral ectatic corneal disorder characterized by inferior corneal thinning, which often leads to irregular astigmatism [1]. It is a progressive, non-inflammatory condition distinguished by a narrow band of thinning that typically spares the limbus [2]. While the inferior cornea is most commonly affected, cases involving superior corneal thinning have also been reported [3]. Fuchs heterochromic iridocyclitis (FHI) is a chronic, typically unilateral anterior uveitis marked by low-grade intraocular inflammation and a generally favorable visual prognosis [4]. Most patients present in their third or fourth decade of life, and the condition shows no strong gender predilection [5]. Clinical features may include glaucoma, heterochromia, cataract, vitritis, and iridocyclitis [6]. This case report highlights a rare co-occurrence of PMD and FHI, underscoring the importance of thorough diagnostic evaluation and tailored management in patients presenting with overlapping corneal and uveal pathology. This case report has been reported in line with the SCARE checklist [7].

## 2 Case report

**Fig. 1:**
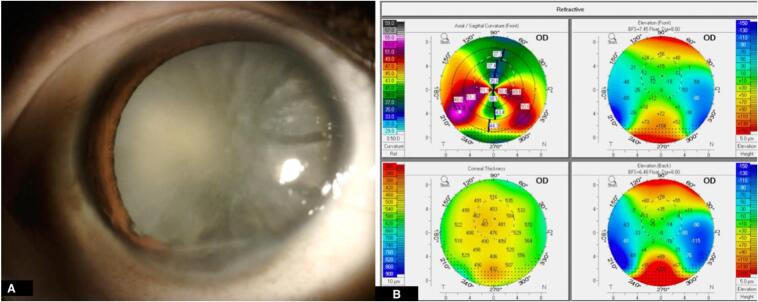
(A) Slit lamp photograph demonstrating a mature cataract in conjunction with a band of peripheral corneal thinning in the inferior quadrant (arrow). (B) Corneal tomography (4-refractive maps) demonstrating the characteristic “crab claw” configuration associated with pellucid marginal degeneration, with thinning predominantly located in the inferior cornea.

**Fig. 2:**
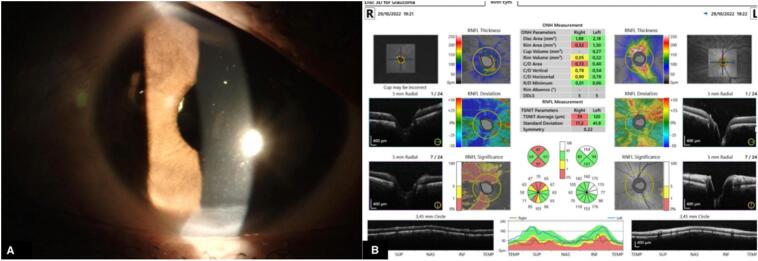
(A) Postoperative slit lamp photograph demonstrating keratic precipitates (arrow) and persistent thinning in the inferior cornea. (B) Optical coherence tomography (OCT) of the optic nerve head demonstrating a cup-to-disk ratio of 0.7.

A 40-year-old woman presented with a gradual, painless decline in vision in the right eye over the preceding two months. Clinical examination revealed an exotropia of 15 prism diopters (PD), inferior corneal thinning and scarring, pigmented keratic precipitates (KPs), and a mature white cataract obscuring the fundus view (Fig. 1, A). Intraocular pressure (IOP) was within normal limits in both eyes. Diagnostic workup included B-scan ultrasonography, which ruled out posterior segment pathology. Corneal tomography (Pentacam® HR, Oculus) confirmed inferior thinning consistent with pellucid marginal degeneration, with a maximum keratometry (Kmax) of 53.9 D, thinnest pachymetry of 458 μm located inferiorly, and a characteristic “crab-claw” pattern on anterior sagittal curvature (Fig. 1, B). The ABCD grading system was not fully applicable, but indices confirmed significant inferior ectasia. Preoperative visual acuity in the affected eye was counting fingers at 1 m and the refraction could not be reliably measured due to the mature cataract. Biometric assessment was performed using the IOLMaster® 700 (Carl Zeiss Meditec). The Barrett Universal II formula was selected, targeting a mild postoperative myopia (−0.50 D) to account for ectatic corneal curvature. Given the irregular astigmatism from PMD and poor reliability of keratometric readings, a toric IOL was not considered. Instead, a monofocal posterior chamber IOL was implanted. The main clear corneal incision was placed temporally at 180°, avoiding the inferiorly thinned corneal zone and minimizing surgically induced astigmatism. No capsular tension ring (CTR) or adjunctive devices were required intraoperatively, as zonular support was deemed adequate. The patient underwent uneventful phacoemulsification with posterior chamber IOL implantation. Intraoperatively, Amsler's sign was noted, characterized by blood reflux into the anterior chamber [4]. This finding is classically associated with FHI and is attributed to rupture of fragile, fine vessels in the anterior chamber angle following sudden intraocular pressure fluctuations such as paracentesis or surgical entry. Importantly, the presence of the Amsler sign reflects vascular fragility in an otherwise open angle and does not imply angle closure. In our case, gonioscopy (Spaeth classification) confirmed that the angle was open in all quadrants (D40r), with approximately 25 % peripheral anterior synechiae (PAS), most pronounced inferiorly. This reconciles the apparent inconsistency between the open angle configuration and the presence of PAS, as the latter likely represents chronic sequelae of longstanding anterior segment inflammation rather than generalized angle narrowing. On postoperative Day 1, the uncorrected distance visual acuity (UDVA) was 20/200, with a clear cornea, quiet anterior chamber, and a well-centered posterior chamber intraocular lens (PCIOL) (Fig. 2, A). Intraocular pressure (IOP) measured 14 mmHg, and the patient was maintained on a topical steroid–antibiotic combination. By Week 1, UDVA had improved to 20/80, with manifest refraction of −0.75/–4.75 × 90° giving a corrected distance visual acuity (CDVA) of 20/80. At the 1-month visit, the refraction remained similar (−0.75/–4.75 × 90°), and CDVA with spectacles was limited to 20/80, consistent with the combined effect of irregular astigmatism, long-standing exotropia, and amblyopia. However, with a hard contact lens (HCL), best-corrected vision improved to 20/40, highlighting the role of rigid lens optics in neutralizing irregular astigmatism in PMD. IOP remained stable at 15 mmHg without antiglaucoma therapy. Anterior segment examination demonstrated a quiet eye with persistent PAS involving approximately 25 % of the angle, predominantly inferiorly. Fundus evaluation revealed a cup-to-disc ratio of 0.7. Optical coherence tomography of the retinal nerve fiber layer (OCT-RNFL) showed borderline thinning, prompting scheduling of visual field testing (Fig. 2, B). The patient was placed on long-term glaucoma surveillance, with planned 6-monthly OCT-RNFL and perimetric evaluations.

## 3 Discussion

PMD is a rare, non-inflammatory ectatic corneal disorder characterized by peripheral inferior corneal thinning. Its subtle clinical presentation often leads to misdiagnosis, especially in early stages, where it may be mistaken for keratoconus or high astigmatism. In this case, PMD contributed to progressive and irregular astigmatism, significantly reducing the patient's visual acuity. Due to its progressive nature, PMD requires regular monitoring. While conservative management with rigid gas-permeable lenses is common in mild cases, advanced or vision-threatening stages may necessitate surgical intervention, such as corneal cross-linking, intracorneal ring segments, or penetrating keratoplasty [1]. Concurrently, the patient presented with signs consistent with FHI—a chronic, typically unilateral, low-grade anterior uveitis. Clinical features including diffuse, stellate KPs, PAS, and a mature cataract suggest a long-standing inflammatory process. Chronic inflammation in FHI can lead to complications such as secondary glaucoma and cataract formation, both of which were evident in this case [7].

The presence of PAS and KPs indicates the chronicity of the inflammatory process, warranting cautious perioperative planning and close postoperative surveillance.

The surgical approach—phacoemulsification with intraocular lens implantation—though standard, posed challenges due to the coexistence of corneal ectasia and uveitic pathology. The altered corneal topography associated with PMD complicates intraocular lens power calculation and surgical wound construction. Additionally, chronic intraocular inflammation increases the risk of postoperative complications such as cystoid macular edema or exacerbation of uveitis. A highly experienced surgical team and preoperative control of inflammation were crucial to achieving a favorable outcome. Postoperatively, the patient demonstrated significant improvement in visual acuity. However, the observation of an enlarged cup-to-disc ratio (0.7) raised concerns about glaucomatous damage—either preexisting or inflammation-induced. Gonioscopy revealed an open-angle configuration, with the presence of PAS suggesting segmental synechial closure, possibly due to synechial formation from prolonged inflammation. This underscores the importance of differentiating between mechanical and inflammatory causes of IOP elevation. Long-term follow-up with regular optic nerve evaluation using optical coherence tomography (OCT) and visual field testing is essential to detect and monitor glaucomatous progression. Moreover, continued use of topical corticosteroids or non-steroidal anti-inflammatory medications is recommended in managing FHI and preventing recurrences or complications.

The simultaneous presentation of PMD and FHI in this patient is exceptionally rare. Although no direct causal relationship has been established in the literature, their coexistence raises intriguing questions about possible shared pathogenic mechanisms. These may include localized immune dysregulation, stromal susceptibility to inflammatory mediators, or genetic predispositions. Further studies are necessary to explore potential associations between corneal ectatic disorders and chronic intraocular inflammation.

## 4 Conclusion

This case highlights the rare co-occurrence of Pellucid Marginal Degeneration and FHI in the same eye, presenting unique diagnostic and surgical challenges. The overlapping manifestations—irregular astigmatism, chronic intraocular inflammation, and advanced cataract—demanded an individualized, multidisciplinary approach. The patient's favorable postoperative outcome demonstrates the importance of tailored surgical planning, inflammation control, and vigilant long-term follow-up.

Clinicians should be aware of this unusual combination, particularly when managing patients with atypical corneal topography and signs of chronic anterior uveitis. Early recognition and careful monitoring of potential complications such as glaucoma and recurrent inflammation are essential to preserve visual function. This case also underscores the need for further research into the possible immunological or genetic associations between corneal ectatic disorders and uveitic entities.

## Author contribution

**AA:** Data collection for cases, Literature review, manuscript revision.

## Patient consent

Written informed consent was obtained from the patient for publication of this case report and accompanying images.

## Ethical approval

No IRB approval is needed for case reports. However, this case report was prepared according to the ethical standards of the human ethics in accordance with the Helsinki Declaration.

## Guarantor

Ali Hendi Alghamdi.

## Research registration number

1. Name of the registry: Research Registry.
2. Unique identifying number or registration ID: researchregistry9653
3. Hyperlink to your specific registration (must be publicly accessible and will be checked): https://www.researchregistry.com/browse-the-registry#home/

## Authorship

Author attests that he meets the current ICMJE criteria for Authorship.

## Funding

This research did not receive any specific grant from funding agencies in the public, commercial, or not-for-profit sectors.

## Conflict of interest statement

Authors have no conflicts of interest in relation to this work.
